# Detection of Potential Chloroplastic Substrates for Polyphenol Oxidase Suggests a Role in Undamaged Leaves

**DOI:** 10.3389/fpls.2017.00237

**Published:** 2017-03-03

**Authors:** Tinne Boeckx, Ana Winters, K. Judith Webb, Alison H. Kingston-Smith

**Affiliations:** Institute of Biological, Environmental and Rural Sciences (IBERS), Aberystwyth UniversityAberystwyth, UK

**Keywords:** polyphenol oxidase, PPO, chloroplast, hydroxycinnamic acids, flavonoids, coumaroyl malate, coumaroyl hexoside

## Abstract

Polyphenol oxidases (PPOs) have a recognized role during pathogen and arthropod attack. As an immediate consequence of such wounding, cellular compartmentation is destroyed allowing the chloroplastic PPO enzyme to interact with vacuolar substrates catalyzing the oxidation of monophenols and/or *o*-diphenols to *o*-diquinones. This ultimately results in a reduction in the nutritional value of wounded tissue through the formation of non-digestible secondary melanin pigments. However, the chloroplastic location of PPO enzyme could indicate a role for PPO in undamaged tissues. In this study, a wild-type red clover population exhibiting high leaf PPO activity had significantly higher yield than a low leaf PPO mutant population while leaf isoflavonoids and hydroxycinnammates (PPO substrates) accumulated at similar levels in these plants. These data suggest that the presence of leaf PPO activity affects plant vigor. Understanding how this advantage is conferred requires knowledge of the cellular mechanism, including intra-organellar substrates. Here we present evidence of candidate PPO substrates within chloroplasts of wild-type red clover, including the monophenolic acid, coumaroyl malate, and low levels of the diphenolic acid, phaselic acid (caffeoyl malate). Interestingly, chloroplastic phaselic acid concentration increased significantly under certain growth conditions. We discuss the implications of this in regard to a potential role for chloroplastic PPO in undamaged leaves.

## Introduction

Polyphenol oxidases (PPOs) catalyze the oxidation of monophenols and/or *o*-diphenols to highly reactive *o*-diquinones. Through secondary reactions, *o*-diquinones can lead to the generation of reactive oxygen species (ROS) and protein complexes, commonly observed as brown melanin pigments (Steffens et al., 1994). The term PPO is often used to refer to either one of its two subclasses; tyrosinases or catecholases. The tyrosinases first catalyze the oxidation of monophenols to *o*-diphenols (monophenolase activity; EC 1.14.18.1) followed by the conversion of *o*-diphenols to *o*-diquinones (catecholase activity; EC 1.10.3.1), whereas catecholases are *o*-diphenol specific. The PPOs characterized to date have mostly been classified as catecholases, however, this could be because the detection of monophenolase activity requires highly specific conditions in comparison to catecholase activity (Yoruk and Marshall, 2003). This would suggest that some catecholases may in fact be tyrosinases in which the monophenolase activity has not yet been observed (Solomon et al., 1996).

Phenolic compounds, and therefore also PPO substrate candidates, are reported to be mainly localized in the vacuole (Yoruk and Marshall, 2003), but their presence has been detected in other subcellular compartments (Moskowitz and Hrazdina, 1981; Shnabl et al., 1986; Weissenböck et al., 1986; Hutzler et al., 1998; Smith-Becker et al., 1998; Chakraborty et al., 2006). Enzymes involved in phenolic biosynthesis such as phenylalanine ammonia-lyase and 4-hydroxycinnamate:coenzyme A ligase have been reported across a number of cellular compartments including in the cytoplasm, nucleus, endoplasmic reticulum (Achnine et al., 2004; Chen et al., 2006). However, while subcellular location varied, phenolic biosynthesis is thought to occur primarily in the cytosol (Zaprometov and Nikolaeva, 2003). Bearing this in mind, the subcellular localization of PPO enzymes and substrates present an intriguing conundrum as mature PPO protein is found loosely attached to the luminal side of the thylakoid membrane in the chloroplastic cellular compartment (Mayer and Harel, 1979; Vaughn and Duke, 1984; Sommer et al., 1994).

An evolved highly conserved targeting mechanism suggests that chloroplastic localization of PPO offers a distinct advantage to plant performance. Given that substrate and enzymes must be co-localized for the catalytic reaction to occur, this suggests one of two options: catalysis occurs only when cell compartmentation is lost or there are, as yet unidentified, PPO substrates present in the chloroplasts. The localization of recognized PPO substrates, *p*-coumaric acid and caffeic acid, within chloroplasts themselves has previously been reported (Satô, 1966; Halliwell, 1975).

PPO activity has most often been associated with biotic stress such as arthropod (Kowalski et al., 1992) or pathogen defense mechanisms (Li and Steffens, 2002; Thipyapong et al., 2004a), both of which cause tissue damage and destroy spatial separation of PPO enzyme and substrate(s), resulting in a decrease in the nutritional value of the tissue due to the formation of *o*-diquinone protein complexes (Felton et al., 1989; Thipyapong et al., 2004a). Conversely, the elucidation of a role for PPOs in chloroplasts that does not involve the destruction of compartmentation has proved more challenging. Notably, despite an apparent performance advantage associated with expression of PPO in healthy tissues (Boeckx et al., 2015a), testing of successive hypotheses concerning a putative role for PPO in protection of photosynthesis from environmental stress have so far proved inconclusive (Tolbert, 1973; Mayer and Harel, 1979; Vaughn and Duke, 1984; Thipyapong et al., 2004b; Boeckx et al., 2015a,b). Identification of chloroplastic substrates for PPO would support the theory that a specific metabolic function is associated with the chloroplastic localization of mature PPO protein.

Potential PPO substrates have been identified within the isoflavonoid, flavanol, flavone, flavonol, and anthocyanin subclasses of flavonoid polyphenols, and within hydroxybenzoic and hydroxycinnamic acid subclasses of phenolic acids (Parveen et al., 2010). Although, these are generally considered to be vacuolar, their subcellular location can be ambiguous. There are numerous studies which demonstrate that chloroplasts accumulate flavonoids (Saunders and McClure, 1976a,b; Agati et al., 2007; Liu et al., 2009), and evidence that these chloroplastic flavonoids can act as PPO substrates has been presented for catechins in tea (*Camellia sinensis*; Subramanian et al., 1999; Liu et al., 2009). In a more recent study two monophenolic compounds, tyrosine and tyramine, have been identified as potential PPO substrates in intact walnut tissue (Araji et al., 2014). Both endogenous accumulation of tyramine in PPO-silenced plants and exogenous application was associated with a spontaneous necrosis phenotype suggesting a role for PPO in regulation of cell death (Araji et al., 2014). Furthermore, the data suggested that PPO regulation of tyramine levels in healthy tissues is mediated by direct involvement in tyrosine catabolic pathways (Araji et al., 2014).

Red clover has been extensively used to study PPO activity (Winters et al., 2003; Sullivan et al., 2004; Lee et al., 2010; Webb et al., 2013). Phaselic acid and clovamide are the main endogenous *o*-diphenol substrates detected in red clover leaves (Winters et al., 2008; Sullivan, 2009). Here, we compared the metabolite profile of whole leaf extracts with those of chloroplasts of wild-type red clover (*Trifolium pratense* L. cv. Milvus) and identified candidate PPO substrate(s) in chloroplasts. We also compared rates of germination, establishment, plant yield and hydroxycinnamate and isoflavonoid accumulation in leaves of glasshouse-grown populations of wild-type and mutant (low leaf PPO activity) red clover. Taken together, the results presented in this study represent a step toward determining whether PPO could have an active role in primary or secondary metabolism in undamaged tissues, and given the potential yield benefit associated with PPO expression, what implications this may have for the exploitation for agricultural gain.

## Materials and methods

### Plant material

Two red clover (*T. pratense* L.) populations, cv. Milvus (wild-type, Aa4381) and a low leaf PPO mutant line (Aa4512; Winters et al., 2008), were sown in John Innes no. 3 compost. The mutant population was a genetic mutant for low leaf PPO, selected from and backcrossed into cv. Milvus (Lee et al., 2004). Leaf PPO activity was confirmed as described elsewhere (Winters et al., 2008).

When plants were grown in a glasshouse, growth conditions were set to a minimum 12 h photoperiod, minimum light intensity of 300 μmol m^−2^ sec^−1^, and day/night temperature of 20/16°C. Plants (8 weeks old) that were acclimatized to a controlled environment in a growth cabinet were maintained under a 16 h photoperiod, 400 μmol m^−2^ sec^−1^ and 20/16°C for 4 weeks prior to treatment.

### Growth measurements and sampling

Seeds of wild-type and low leaf PPO mutant plants were sown 4 per pot) and grown in a glasshouse. At 3 weeks, measurements of germination rate, number of shoots per plant, and number of leaves per shoot were taken (*n* = 60) before plants were reduced to 1 plant per pot. Plant height was recorded at intervals up to 8 weeks after sowing (*n* = 15). Height measurements were taken from the base of the plant to the node of the uppermost leaf on the longest shoot. Dry matter yield of leaf and stem material was measured separately and whole leaf samples were flash frozen and stored at −80°C or freeze-dried and milled for isoflavonoid and hydroxycinnamate analysis.

For a comparison and analysis of isoflavonoids and hydroxycinnamates in whole leaves, leaf samples were taken from glasshouse grown mature, 8 week old wild-type and mutant (low leaf PPO) red clover plants. These were freeze-dried, milled, and weighed (50 ± 2.5 mg samples) and then extracted for isoflavonoids and hydroxycinnamic acids as described below.

Equivalent plants were acclimatized to a controlled environment (16 h photoperiod, 400 μmol m^−2^ sec^−1^, 20/16°C) for 4 weeks and used for a more detailed analysis of the effect of light on phaselic acid (caffeoyl malate) and coumaroyl malate in isolated chloroplasts from low leaf PPO mutant and wild-type plants. Randomly selected leaves were sampled separately (*n* = 6) for whole leaf extraction (6 × 1 g samples) and chloroplast purification (6 × 10 g samples). Samples were harvested from six 12 week old plants in a two phase cross over design; plants were either illuminated or kept in dark for a minimum of 7 and 16 h, respectively. After the first sampling in the light or dark, the same plants were transferred to the opposite conditions (i.e., light to dark and dark to light). The remaining leaves were harvested after the equivalent time in the light or dark. Thus, 2 × 3 light adapted plants were sampled 7 h into a light period while 2 × 3 dark adapted samples were harvested after 16 h darkness at room temperature; the difference between plants was the level of stress caused by removal of leaves for harvest at the first sampling.

### Chloroplast preparation

Chloroplasts were isolated from leaves (6 × 10 g samples) essentially as described by Mills and Joy (1980) except that initial homogenization was performed with a hand-held blender (Stabmixer SM3, Primo, Antwerp, Belgium) for three cycles of 5 s, with a 10 s pause between each. Intact chloroplasts were collected by centrifugation through 40% Percoll, the pellet resupended in <1.0 ml buffer A (Mills and Joy, 1980) and intactness confirmed by microscopy under UV illumination (Olympus BH2-RFCA T3; Olympus, Tokyo, Japan). The remainder was centrifuged for 1 min at 4°C and 2,500 × g to remove any remaining Percoll and the pellet was resuspended in 300 μL pre-cooled lysis buffer C.

### Extraction of isoflavonoids and hydroxycinnamic acids

Isoflavonoids and hydroxycinnamic acids were extracted from the three sample types (freeze-dried leaf samples, whole leaf samples, and lysed chloroplast suspension) in methanol. The stability of biochanin and formononetin at 90°C for 5 min has previously been demonstrated (Stintzing et al., 2006). The freeze-dried leaf samples (50 ± 2.5 mg samples) were extracted x1 in 0. 5 mL 70% methanol at 90°C for 5 min followed by x2 in 0.5 mL 70% methanol at room temperature. The whole leaf (1 g) samples were ground in liquid nitrogen pre-cooled pestle and mortars to a fine powder to which 10 mL of 70% methanol at 90°C was added for 5 min. Lysed chloroplast samples were brought to a final concentration of 70% methanol by addition of 100% methanol. Aliquots of methanolic extracts from chloroplast and whole leaf samples were taken to assess chlorophyll content according to Arnon (1949). The remainder of the samples were treated to decrease the methanol concentration to <10% by either rotary evaporation or dilution with deionized water. These were then applied to pre-conditioned (4 mL of 100% methanol) and equilibrated (4 mL of 5% acetic acid) Waters Sep-Pak Vac RC (500 mg) C_18_ reverse-phase extraction cartridges (Waters, Milford, USA) on a vacuum manifold. After washing with 5% acetic acid, the lower-polarity analytes of interest were eluted with 100% methanol. These fractions were evaporated to complete dryness, the residue re-dissolved in 80 μL of 100% methanol and insoluble material removed by centrifugation at 14,100 × g for 4 min. The supernatant was retained for metabolite profiling by HPLC-PDA-ESI-MS^n^.

### Isoflavonoid and hydroxycinnamic acid profiling by HPLC-PDA-ESI-MS^n^

HPLC/MS^n^ analysis of chloroplast and total leaf extracts was carried out with a Thermo Finnigan LTQ system (Thermo Electron Corporation, USA). The system was fitted with a Waters C18 reverse phase Nova-Pak column (4 μM, 3.9 × 150 mm; Waters, Milford, USA) which was maintained at 30°C. Injection volumes of 10 μL were separated by HPLC and the UV absorbance was recorded (240–400 nm). The mobile phase consisted of 0.1% formic acid in deionized water (mobile phase A) and 0.1% formic acid in methanol (mobile phase B). The percentage of mobile phase B was increased linearly from 0 to 65% over 45 or 60 min, with 0.1 mL min^−1^ going to the mass spectrometer. Mass spectra were obtained using a Finnigan LTQ linear ion trap instrument with an ESI source (Thermo Electron Corporation, USA). Nitrogen was used as the sheath (40 units) and auxiliary gas (25 units) and helium was used as the collision gas. Spectra were obtained in negative ionization mode with the following settings: spray voltage, 4 kV; capillary temperature, 320°C; capillary voltage, −45 V; tube lens, −93.1 V. In positive ionization mode the settings were as follows: spray voltage, 4 kV; capillary temperature, 320°C; capillary voltage, 44 V; tube lens, 85 V. Generally collision energy of 35% was used, however, where limited ion fragmentation was observed collision energy was raised to 70%.

Compounds were identified with based on the characteristic MS^n^ fragmentation patterns of isoflavonoid (daidzein, genistein, biochanin A, and formononetin) and hydroxycinnamic acidse (caffeic acid, p-coumaric acid, and ferulic acid) standards. Some peaks eluting close together were observed to have the same mass and fragmentation pattern and were therefore designated with the same ID number. This may be due to the separation of the dissociated and non-dissociated forms of acids. Metabolite abundance was determined by integration of peak area (Qual Browser of the Xcalibur 1.4 software; Thermo Electron Corporation, USA). These measurements were then expressed on the basis of the chlorophyll content in order to predict the likelihood of chloroplastic subcellular localization. The compound was viewed as chloroplastic and not due to contamination if ≥0.1% of the whole leaf concentration was detected within chloroplasts. If the percentage was ≤0.01%, then the compound was assumed to accumulate mainly outside of the chloroplast.

### Statistical analysis

Significant differences between the growth parameters of the two varieties were analyzed by repeated measurements by REML; other growth and metabolite data were analyzed using the student *T*-test and ANOVA, GenStat 14th Edition.

## Results and discussion

**Figure 1:**
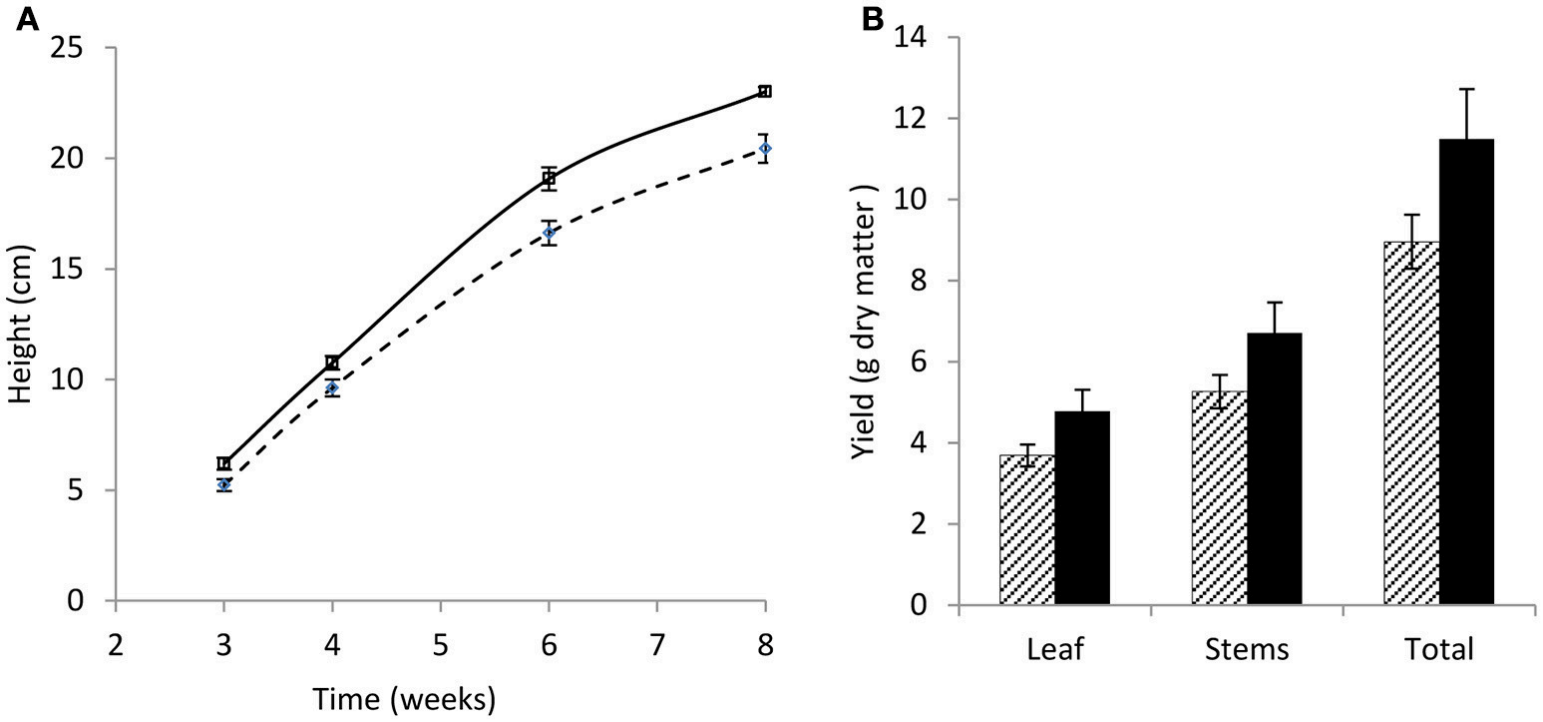
**Growth rate and yield of wild-type and low leaf PPO mutant red clover over 8 weeks. (A)** Growth rate showing plant height (cm), and **(B)** yield of leaf, stem, and total leaf and stem combined (g dry matter). Wild-type (WT) solid; low leaf PPO mutant (MUT) dashed; data show average data with error bars ± SEM (*n* = 15); **(A)** *p* < 0.001; **(B)** nsd.

Over 8 weeks, the wild-type red clover population had the same overall growth rate but a significant increase in plant height (*p* < 0.001; Figure 1A) compared with the low PPO mutant population; final dry matter yield in both stem and leaf was also higher in the wild-type population (Figure 1B). A difference in yield has previously been reported in field-grown plants (Boeckx et al., 2015a). The population difference was clearly observed in the first 3 weeks of their growth; while seed germination was similar, seedling establishment, as indicated by the number of shoots per plant and number of leaves per shoot, was significantly higher (*p* < 0.001) in the wild-type red clover plants as compared with the low leaf PPO mutant population (Table 1). These data indicate that the major significant differences in the activities of leaf PPO between the mutant and wild-type populations (Table 2) subtly affects both plant viability and growth of red clover (Winters et al., 2008). Further analysis is needed to confirm a genetic relationship between plant yield and leaf PPO activity.

**Table 1:** **Seed germination and size of wild-type and low PPO mutant red clover**.

	**Germination**	**No. shoots**	**No. leaves**
Wild-type Mutant	75 ± 4.88	7.87 ± 0.71	16.60 ± 1.46
	60 ± 7.64	4.53 ± 0.56	9.07 ± 1.13
	*p* = 0.109	*p* = <0.001	*p* = <0.001

*Average seed germination (%), number of shoots per plant, and number of leaves per shoot after 3 weeks growth. Error bars ± SEM (n = 60)*.

**Table 2:** **Leaf PPO activity established wild-type and mutant plants grown in glasshouse**.

**Plant**	**Leaf PPO activity (Δ_420_ OD/g F Wt)**		
	**Active**	**Latent**	**Total**
Wild-type Mutant	137.2 ± 56.78	381.8 ± 77.15	518.9 ± 109.02
	4.9 ± 2.92	11.8 ± 2.74	16.7 ± 5.52
	*p* = 0.048	*p* = 0.001	*p* = 0.002

*Average active, latent, and total leaf PPO activity after 3 weeks growth. Error bars ± SEM (n = 5)*.

**Figure 2:**
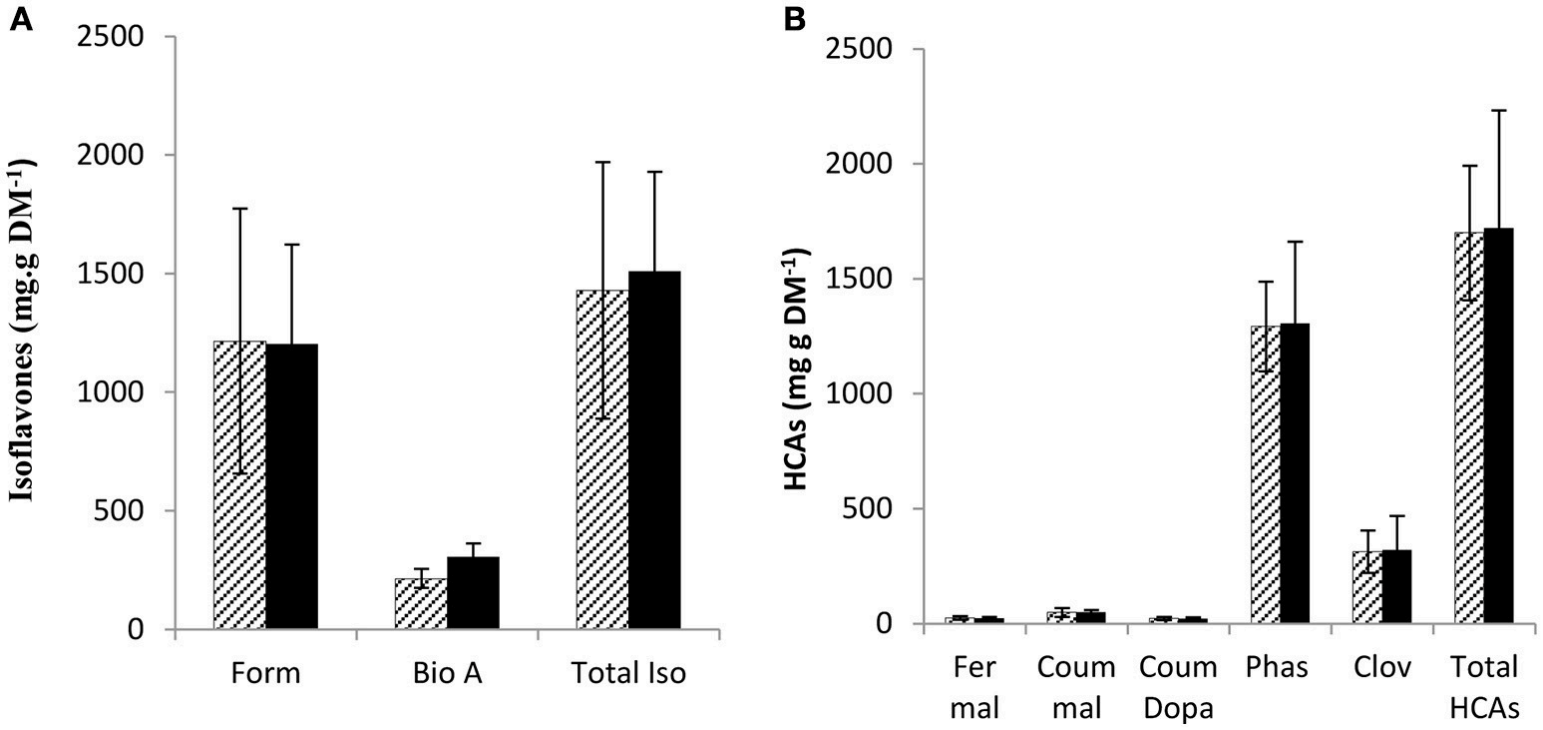
**Soluble phenol content in leaves of wild-type and low PPO mutant red clover after 8 weeks**. Wild-type (WT) solid; low leaf PPO mutant (MUT) dashed line, **(A)** isoflavonoids: formononetin (Form), biochanin A (Bio A) and total isoflavonoid content (Tot Iso) and **(B)** hydroxycinnamic acids: (HCA) feruloyl malate (Fer mal), coumaroyl malate (Coum mal), coumaroyl Dopa (Coum Dopa), phaselic acid (Phas), clovamide (Clov), total hydroxycinnamates (Total HCAs). Error bars ± SEM (*n* = 15); **(A,B)** nsd.

PPO substrates identified so far are polyphenols, secondary metabolites with antioxidant capacity (Pietta, 2000; Cheynier, 2005; Parveen et al., 2010). Although, leaf PPO activity differed significantly between mutant and wild-type leaves (Table 2) no significant difference was observed in either leaf isoflavonoid (Figure 2A) or hydroxycinnamic acid (Figure 2B) abundance in leaf tissues.

### Candidate red clover PPO substrates

**Figure 3:**
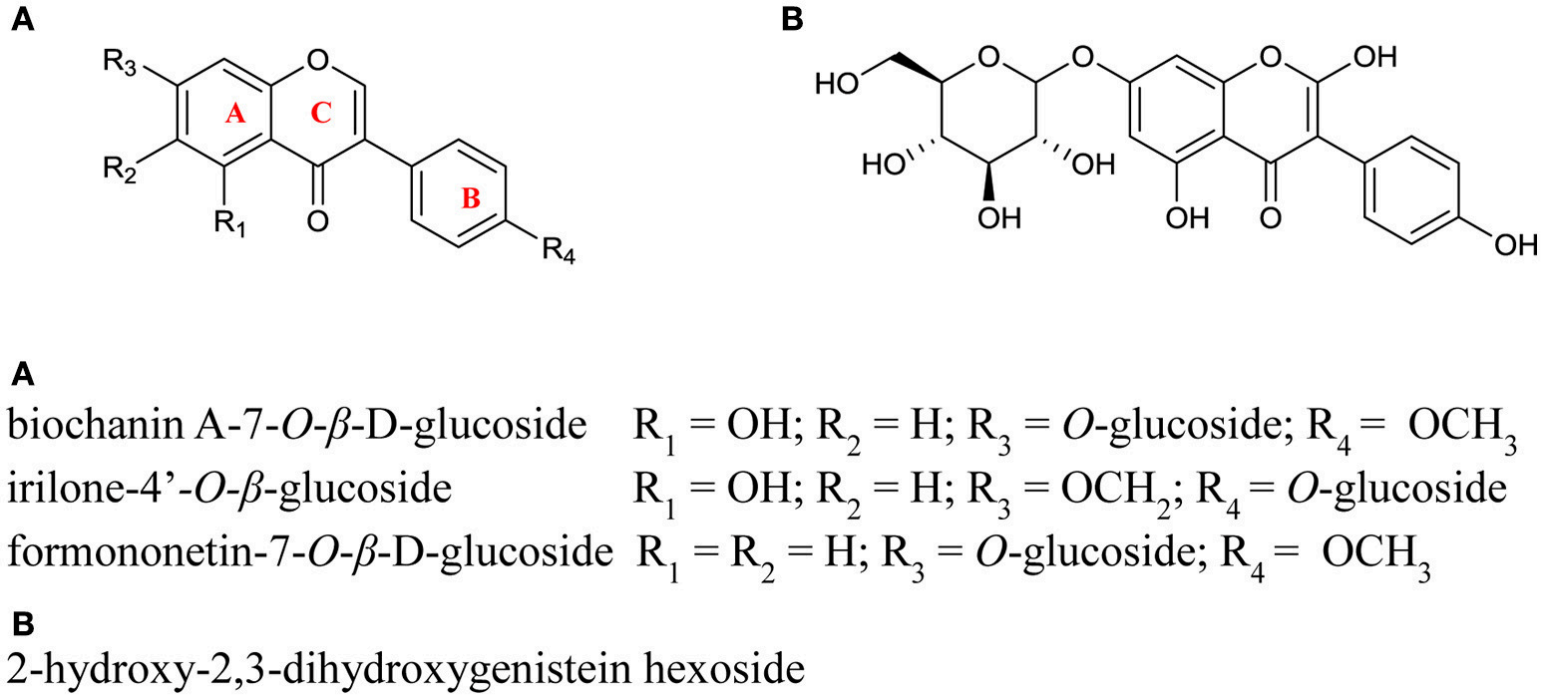
**Structures of the isoflavonoid conjugates identified in red clover leaf extracts**. Positioning of the glycone groups were adapted from Lin et al. (2000) and were not confirmed in this study.

**Figure 4:**
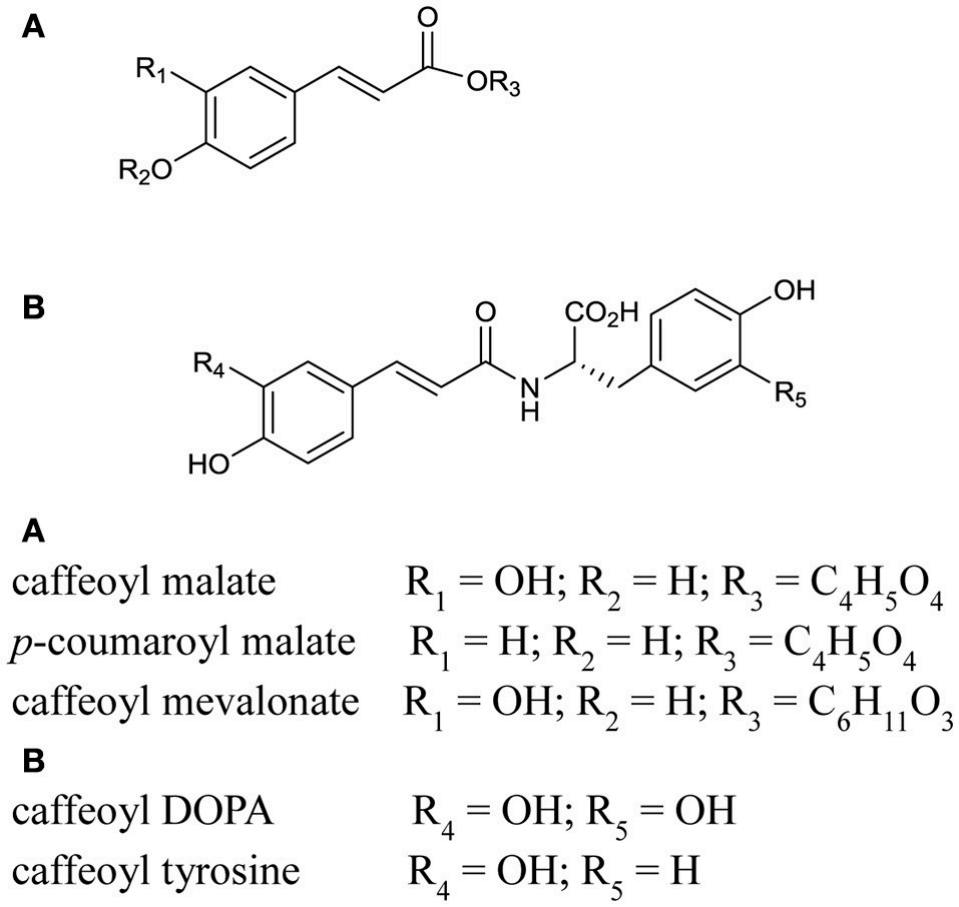
**Structures of tentatively identified hydroxcinnamic acid conjugates and likely PPO substrate candidates found in red clover leaf extracts**. The positioning of the hydroxyl, glycoside and amino acid groups was based on Parveen et al. (2010).

**Figure 5:**
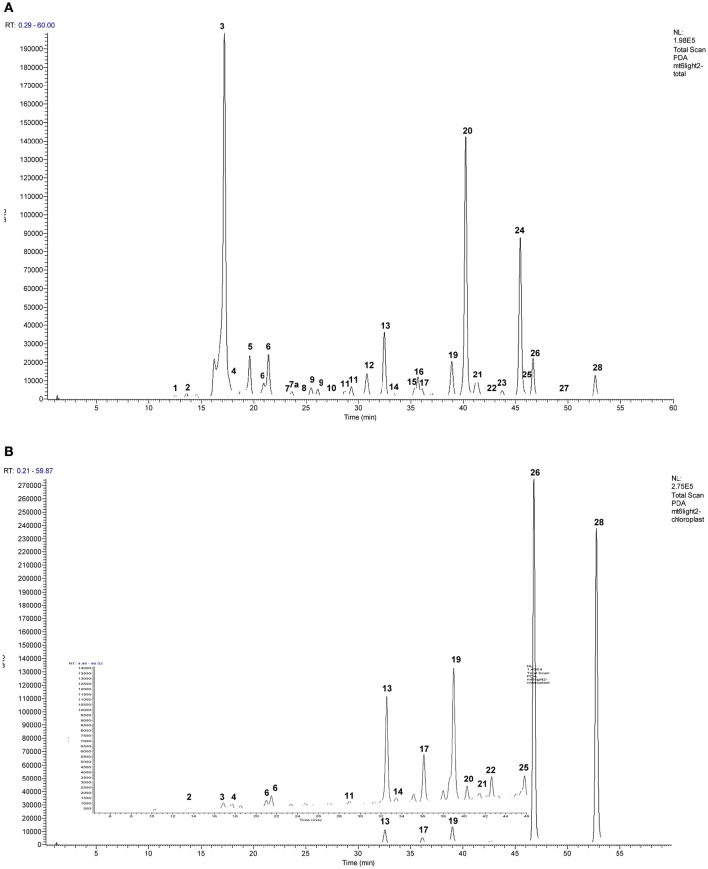
**Representative HPLC/UV chromatograms (240–400 nm) of the phenolics in representative purified (A)** whole leaf extracts and b) chloroplasts of red clover. Final concentration of the extracts shown was 0.1 μg total chlorophyll (Chl_a_ + Chl_b_) μL^−1^ extract of which 10 μL was injected. Panel **(B)** has an enlarged insert to show smaller peaks more clearly. Table 4 shows detail of identified peaks 1–28: 1, caffeic acid; 2, 2-hydroxy-2,3-dihydrogenistein hexoside; 3, caffeoyl malate (phaselic acid); 4, unidentified compound; 5, caffeoyl DOPA (clovamide); 6, coumaroyl malate; 7, coumaroyl DOPA; 7a caffeoyl tyrosine; 8, feruloyl DOPA; 9, caffeoyl mevalonate (tentative); 10, coumaroyl tyrosine; 11, quercetin hexoside; 12, quercetin hexoside malonate; 13, formononetin hexoside, formic acid adduct; 14, kaempferol hexoside; 15, kaempferol hexoside malonate; 16, formononetin hexoside, sodium formate adduct; 17, irilone hexoside, formic adduct; 18, irilone conjugate formic adduct; 19, biochanin A hexoside, formic adduct; 20, formononetin hexoside malonate; 21, isomer of peak (19), formic acid adduct; 22, pratensein; 23, formononetin hexoside succinate; 24, biochanin A, hexoside malonate; 25, pseudobaptigenin; 26, formononetin; 27, biochanin A conjugate; 28, biochanin A.

Known PPO substrates belong to just two classes of polyphenols: flavonoids and phenolic acids (Parveen et al., 2010). Flavonoids have a fifteen carbon structure, comprising two aromatic rings (A- and B-rings) linked by a three carbon bridge (C-ring; Figure 3). Subclasses of flavonoids (isoflavonoids, flavones, flavonols, anthocyanins, and flavanols) are known to include some PPO substrates however, an even larger number are hydroxycinnamates, a subclass of phenolic acids with a characteristic C_6_-C_3_ structural skeleton (Figure 4); these include the known red clover PPO substrates phaselic acid (caffeoyl malate) and caffeoyl DOPA (clovamide) (Sullivan and Hatfield, 2006; Winters et al., 2008). A comprehensive tandem MS analysis was performed in order to identify new red clover PPO substrate candidates located in leaves and chloroplasts (Figure 5).

PPO can utilize flavonoids as substrates where one or more hydroxyl groups are present on the B ring (Martinez and Whitaker, 1995; Parveen et al., 2010). In this study isoflavonoids were identified as both the hexoside conjugated and unconjugated forms of formononetin and biochanin A, irilone hexoside, and 2-hydroxy-2,3-dihydrogenistein hexoside (see Figure 5 and Table 3). As the functional groups on the B ring of the major isoflavonoids detected (biochanin A and formononetin) are restricted to a single methoxy group, and there is no evidence that red clover leaf PPO enzymes can catalyze oxidation of unconjugated formononetin, biochanin A, or genistein it is considered unlikely that the isoflavonoids identified in this study are endogenous red clover PPO substrates.

**Table 3:** **Tandem MS fragmentation (MS^**2**^–MS^**4**^) patterns and characteristic UV spectra for the compounds indicated in Figures **5A,B****.

**Peak no**.	**MS t_*R*_ (min)**	**m/z [^(−)^−ve or ^(+)^−ve ion mode]**	**MS^2^ fragmentation**	**MS^3^ fragmentation (MS^4^ fragmentation) of underlined fragment**	**λ_max_ (nm)**	**Identification**
1	12.5	179^(−)^	**179**, 135, 136, 180		328, 280sh	Caffeic acid^b^
2	13.5	449^(−)^	**287**, 421, 259	**259**, 243, 287, 201, 269 (**259**, 215, 125) (**243**, 199, 215, 149)	283	2-hydroxy-2,3-dihydrogenistein hexoside^c^
3	16.2/17.2	295^(−)^	**179**, 133, 135	**179**, 135	328, 301sh	Caffeoyl malate (phaselic acid)^a^
4	17.7	435	**273**		275	Unidentified compound
5	19.6	358^(−)^	**222**, 178, 161, 223, 179, 135	**222**, 178	290, 321	Caffeoyl DOPA (clovamide)^a^
6	20.9/21.5	279^(−)^	**163**, 133	**163**, 119	313	Coumaroyl malate^b^^,^ ^d^
7	23.1/23.9	342^(−)^	**206**, 207, 163, 161, 135, 119	**163**, 119	N.D	Coumaroyl DOPA^*b??^
8	24.7	372^(−)^	**250**, 222, 236, 218, 178, 340, 161, 372, 135, 109, 193, 140, 328, 296	**250**, 218, 174, 109, 147 (222) **178**, 205, 179 **193**, 161	293sh, 325	Feruloyl DOPA^b^
9	25.4/26.2/	309^(−)^	**179**, 161, 277, 135	**179**, 135	302sh, 328	Caffeoyl mevalonate (tentative)^b^
10	26.9	326^(−)^	**206**, 282, 147, 163, 134, 146, 145, 119	**163**, 119	N.D.	Coumaroyl tyrosine^b^
11	29.4	463	301, 179,151, 343			Quercetin hexoside^c^
12	30.8	549^(−)^ 505^(−)^, frag.	505 **301**, 463, 445	(301) **151**, **179**, 193, 211, 192, 163, 213 (**151**, 107) (**193**, 149)	N.D.	Quercetin hexoside malonate^c^
13	32.6	475^(−)^	**267**, 429	**267**, 252	248, 296sh	Formononetin hexoside, formic acid adduct^c^
14	33.4	447^(−)^ 285^(−)^, frag.	**285**, 327, 255, 179, 151		N.D.	Kaempferol hexoside^3^
15	35.4	533	**489**, 487, 285, 179, 151		ND	Kaempferol hexoside malonate^3^
16	35.8	515^(−)^ 267^(−)^, frag.	**447**, 267		258, 297	Formononetin hexoside, sodium formate adduct^c^
17	36.1	505^(−)^	**459**, 297	(459) **297**, 415, 459 **297**, 269	N.D.	Irilone hexoside, formic adduct^3^
18	37.7	547^(−)^	**501**, 297, 471, 447	297	N.D.	Irilone conjugate formic adduct^c^
19	39.0	491^(−)^	**283**, 445	268	260, 326sh	Biochanin A hexoside, formic adduct^c^
20	40.3	517^(+)^	**269**, 431		249, 296sh	Formononetin hexoside malonate^c^
21	41.5	491^(−)^	**445**, 283		N.D.	Isomer of peak (**19**), formic acid adduct^c^
22	42.6	299^(−)^	**284**			Pratensein^c^
23	43.8	531^(+)^	**269**, 449, 431		260, 296sh	Formononetin hexoside
		575^(−)^, adduct	**529**, 267	**267**, 252, 253		succinate^c^
24	45.5	533^(+)^ 283 ^(−)^, frag.	**533**, 285, 447 **283**, 268		260, 325sh	Biochanin A hexoside malonate^c^
25	45.8	281^(−)^	**281**,			Pseudobaptigenin^c^
26	46.6	267^(−)^	**267**, 252, 253	252	301	Formononetin^c^
27	49.7	547^(+)^			260, 328sh	Biochanin A
		591^(−)^, adduct	**545**, 283	**283**, 268		conjugate^c^
28	52.6	283^(−)^	**283**, 268	268	260, 326sh	Biochanin A^c^

*MS retention time (t_R_). Unless marked by “~,” the average t_R_ across all genotype × extract × treatment groups' MS chromatograms is displayed. “Not detected (N.D.)” refers to either (1) the inability to relate a UV peak to the t_R_ or an impure peak due to the presence of several, generally equally abundant, compounds at the particular t_R_. Mass range search for the parent ion in both positive and negative mode, and subsequent MS^2^ dependent scan, meant that in some cases it was possible to detect where unstable ions had fragmented under electrospray conditions resulting in co-elution of the aglycone with the parent ion. “frag.” refers to ions which are evidently fragmented from a parent ion in full MS*.

a *main leaf PPO substrates*,

b *potential leaf PPO substrates*,

c *flavonoids synthesized in chloroplasts*,

d *potential PPO substrates monophenol substrate detected in chloroplasts. Values in bold indicate the base peak that are fragmented in MS^3^ or MS^4^*.

The hydroxycinnamic acids, phaselic acid (caffeoyl malate; peak 3) and caffeoyl L-3,4-dihydroxyphenylalanine (N-caffeoyl L-DOPA or clovamide; peak 5) are the main PPO substrates in red clover leaves (Sullivan and Hatfield, 2006; Winters et al., 2008) and were clearly identified in this study (Figure 5 and Table 3). In addition caffeoyl conjugates, caffeoyl mevalonate (peak 9, Figure 5A) and caffeoyl tyrosine (peak 7a, Figure 5A) were also tentatively identified and these compounds have very similar structural characteristics to phaselic acid and caffeoyl DOPA respectively (Figure 4). Hence caffeoyl mevalonate and caffeoyl tyrosine are potential endogenous substrates for red clover PPO (Parveen et al., 2010). In addition to the caffeoyl esters and amides, feruloyl DOPA (peak 8; Table 3) was also observed. Because red clover PPOs have also been reported to oxidize the *o*-diphenol DOPA (Parveen et al., 2008; Winters et al., 2008), feruloyl DOPA (peak 8) is another endogenous red clover PPO substrate candidate.

The hydroxycinnamic acid structures thus far discussed are typical of candidate PPO substrates because of their *o*-diphenolic structure with readily oxidizable OH-groups (Martinez and Whitaker, 1995; Parveen et al., 2010). The *o*-diphenolic or catecholase activity is characteristic for PPOs and is the catalytic activity most frequently discussed in the literature. However, some PPOs also have monophenolase activity (Steffens et al., 1994). This has been demonstrated for red clover leaf extracts using monophenolic *p*-coumaric acid and a catalytic amount of *o*-diphenol (Winters et al., 2003). *p*-coumaric acid conjugated with an organic acid (peak 6, coumaroyl malate), a glycoside (coumaroyl hexoside), and an amino acid conjugate (peak 10, coumaroyl tyrosine) were observed in our extracts (Figure 5 and Tables 3, 4).

**Table 4:** **Proportion of red clover isoflavonoids and hydroxycinnamic acid conjugates, present in whole leaves and chloroplasts compiled from separate comparative chromatograms**.

**Phenolic accumulation**	**Peak ID**	**Tentative identification**	**Percent**	**Location/potential role**
Chloroplasts	26	Formononetin	25.0	Flavonoid synthesized in chloroplasts
		Coumaroyl hexoside	20.0	Potential leaf PPO substrate
	28	Biochanin A	14.3	Flavonoid synthesized in chloroplasts
	17	Irilone hexoside	5.88	Flavonoid synthesized in chloroplasts
	19	Biochanin A hexoside	3.45	Flavonoid synthesized in chloroplasts
	13	Formononetin hexoside	1.89	Flavonoid synthesized in chloroplasts
	2	2-hydroxy-2,3-dihydrogenistein hexoside	0.31	Flavonoid synthesized in chloroplasts
	6	Coumaroyl malate	0.15	Potential PPO monophenol substrate
External to chloroplasts	1	Caffeic acid	0.01	
	10	Coumaroyl tyrosine	0.01	Potential leaf PPO substrates
	3	Caffeoyl malate (phaselic acid)	0.002	Main leaf PPO substrates
	5	Caffeoyl 3,4-dihydroxyphenylalanine DOPA (clovamide)	0.0004	Main leaf PPO substrates
	7^a^	Caffeoyl tyrosine	n/d	Potential leaf PPO substrates
	8	Feruloyl DOPA	n/d	Potential leaf PPO substrates
	9	Caffeoyl mevalonate	n/d	Potential leaf PPO substrates

*Illuminated, young fully expanded leaves from wild-type red clover (T. pratense cv. Milvus; n = 3) were sampled for chloroplast and whole leaf extracts. Molecular weight (MW) was detected by mass spectrometry in −ve ion mode. Peak areas were collected from full MS spectra and normalized per μg total leaf chlorophyll (Chl_a+b_). (n/d) not detected in chloroplasts*.

An early report by Satô (1966) showed a copper-containing enzyme catalyzing the oxidation of *p*-coumaric acid to caffeic acid in isolated chloroplasts of several plant species including *Saxifraga stolonifera*; this was subsequently found to express PPO enzyme activity (Jeong et al., 2005). Moreover, in a study with chloroplasts isolated from *Beta vulgaris*, Halliwell (1975) observed that hydroxylation of *p*-coumaric acid only occurred in the light unless a reductant such as ascorbate, NADH, or NADPH was included. On the basis of these observations they proposed that this reaction is catalyzed by PPO monophenolase activity which is dependent on a reducing environment. Thus, the identification of coumaroyl esters in red clover leaf extracts could be indicative of novel, potentially chloroplastic, monophenolic PPO substrate candidates, and a precursor for phaselic acid.

### Phenolic compounds in chloroplasts

In order to determine the likelihood that identified candidates are PPO substrates in intact tissue, the hydroxycinnamate profile of whole leaf extracts and purified chloroplasts was compared (Figures 5A,B). The profile obtained from the chloroplast extracts was distinct from that from whole leaf extracts with six isoflavonoids and two potential PPO substrates (coumaroyl hexoside and coumaroyl malate) showing enrichment in the plastid fraction (Table 4).

Chloroplast preparations were examined microscopically prior to lysis to confirm that the chloroplasts were mainly intact. However, we acknowledge that the integrity of the membrane could be breached allowing bidirectional flow of constituents between chloroplast and cytosol. However, comparison of chromatograms from chloroplast and whole leaf extracts showed that a major peak corresponding to phaselic acid in total leaf extracts (RT 17.1 min) was only just detectable in the chloroplast profile (Figure 5B, and Table 3). As phaselic acid has been previously shown to be present in leaves of red clover (Sullivan and Hatfield, 2006; Winters et al., 2008; Sullivan, 2009) this indicates that the lysed chloroplast preparations were free from contamination and could be used to identify candidate PPO substrates specifically targeted to the chloroplasts.

**Figure 6:**
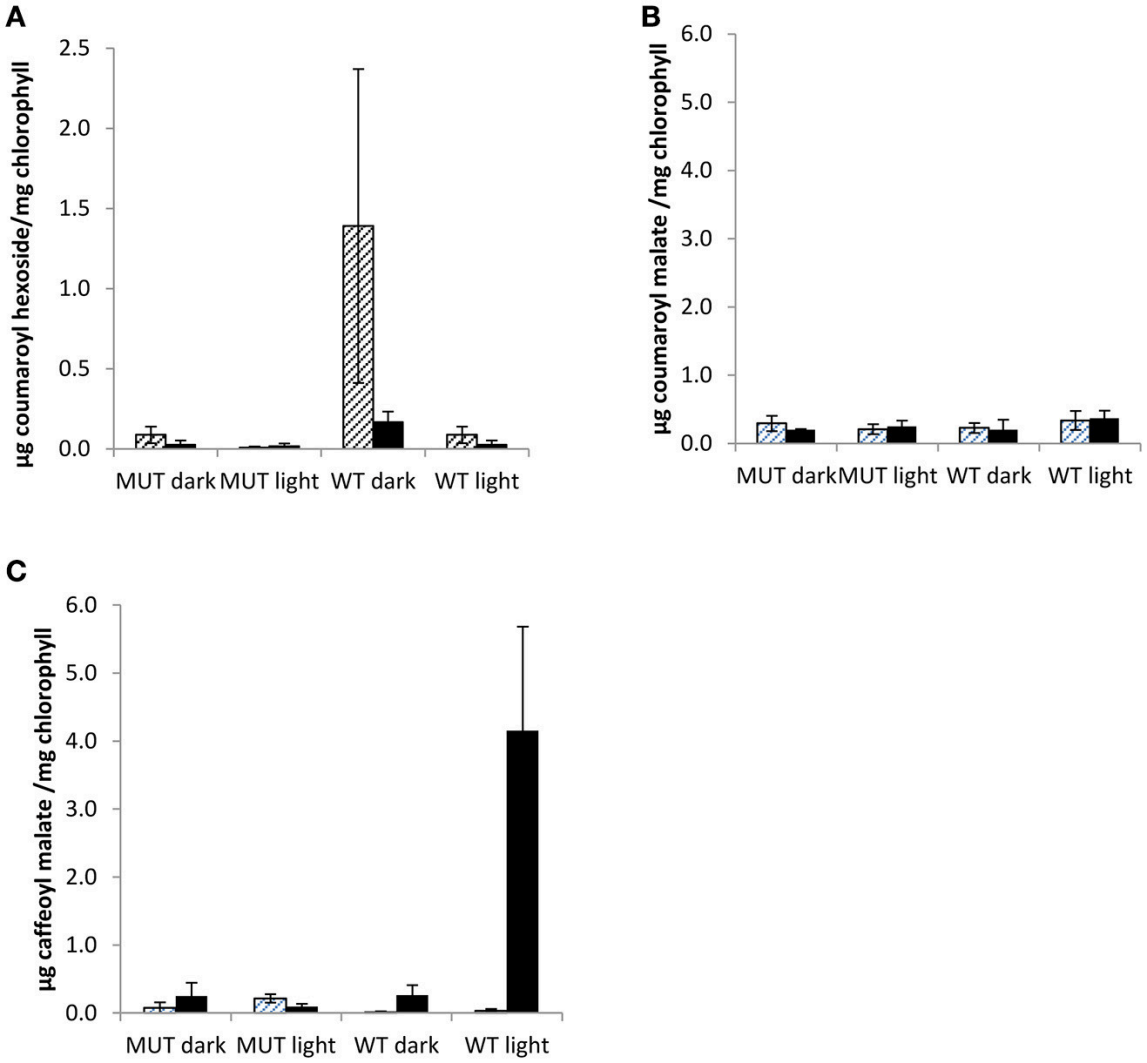
**Chloroplastic phenolics in low leaf PPO mutant and wild-type plants grown in a two phase cross over design**. Chloroplastic phenolics **(A)** coumaroyl hexoside, **(B)** coumaroyl malate, and **(C)** caffeoyl malate (phaselic acid) detected in first phase (cross hatching) and second phase (stippled) of two phase cross over design. MUT, Mutant; WT, wild-type; error bars ± SEM (*n* = 3).

Coumaroyl malate, monophenolic PPO substrate candidate, appeared to be enriched in red clover chloroplasts (Figures 5A,B and Table 4), along with coumaroyl hexoside (Figure 6 and Table 4). By contrast, the known *o*-diphenolic red clover PPO substrates phaselic acid and clovamide are predominantly located in extra-chloroplastic compartments, with on average only 0.002 and 0.0004%, respectively of the total leaf concentration being detected (Table 4) in chloroplasts isolated from unstressed leaves. In addition, other candidate substrates, such as caffeoyl tyrosine (peak 7a), feruloyl DOPA (peak 8), and caffeoyl mevalonate (peak 9) were all below detection limits in red clover chloroplasts (Table 4). Based on these data, caffeoyl derivatives that would be generated from PPO catalyzed oxidation of coumaroyl conjugates do not accumulate in chloroplasts but are exported to the cytoplasm. Since PPO is generally present in a latent inactive form, it is possible that evidence of such activity can only be observed during periods of stress.

Purified red clover chloroplast extracts were particularly abundant in isoflavonoids (Table 4), most notably the aglycone forms of formononetin and biochanin-A. This observation is in agreement with the literature which documents the presence of flavonoids in the chloroplast of the Atlas 68 barley variety (Saunders and McClure, 1976b), the green olive tree (*Phillyrea latifolia*; Agati et al., 2007), tea (*C. sinensis*; Liu et al., 2009) and in 23 out of 25 other vascular plant species surveyed (Saunders and McClure, 1976a). The role of flavonoids in chloroplasts remains unclear, although they may be required for the protection of the plant's active photosynthetic machinery. Barley plastids selectively accumulated the C-glycosyl flavone, saponarin, and since this accumulation is far-red reversible, it is possibly controlled by phytochrome (Saunders and McClure, 1976b). Agati et al. (2007) found that photo-induced generation of ^1^O_2_ was inversely related to the content of flavonoids in the mesophyll cells of *P. latifolia* leaves. These data suggest a complementary protective role for flavonoids in photosynthetically active chloroplasts under high light stress.

### The role of PPO catalysis in the chloroplast

The presence of PPO in the chloroplast has long prompted the question of why PPO resides in this particular cellular compartment, as physical separation from its substrates can also be achieved in the cytosol, and therefore if there is indeed a role for PPO independent of tissue damage (Yoruk and Marshall, 2003; Thipyapong et al., 2004b; Boeckx et al., 2015b). Many phenolics are antioxidants (Pietta, 2000; Parveen et al., 2010), acting as radical scavengers (Neill and Gould, 2003; Agati et al., 2007) and photochemical energy dissipaters (Grace and Logan, 2000; Neill and Gould, 2003) which could contribute to the protection of the chloroplast against stress.

In this study, phenolic contents of purified chloroplasts were analyzed from 12 week old mutant and wild-type plants in a two phase cross over design. The chloroplastic metabolites, coumaroyl hexoside, coumaroyl malate, and phaselic acid (caffeoyl malate), were detected in both phases of the cross over design (Figure 6).

**Figure 7:**
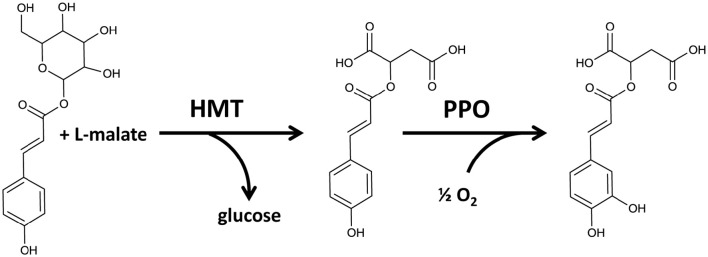
**Proposed pathway for conversion of coumaroyl hexoside to coumaroyl malate catalyzed by hydroxycinnamoylglucose:malate hydroxycinnamoyltransferase (HMT) (Lehfeldt et al., **2000**) and the subsequent conversion of coumaroyl malate to caffeoyl malate (phaselic acid) catalyzed by PPO**.

In mutant plants, none of the three chloroplastic metabolites differed significantly (Figure 6). However, in wild-type purified chloroplasts both coumaroyl hexoside and phaselic acid accumulated as compared with the mutant, although during different phases of the experimental design (Figure 6), and this accumulation appeared to be light dependent. Coumaroyl hexoside was detected at elevated levels in the dark in both phases of the cross over design; though it was significantly higher in the first phase only (*p* < 0.05; Figure 6A). No differences were observed in accumulation of coumaroyl malate (Figure 6B), while an increase in phaselic acid, detected in the second (light) phase (*p* < 0.01; Figure 6C), strongly indicated that this was an effect of PPO activity in illuminated, wild-type chloroplasts, consistent with the findings of Halliwell (1975). A proposed pathway for the conversion of coumaroyl hexoside to phaselic acid is presented in Figure 7. In this the light independent conversion of coumaroyl hexoside to coumaroyl malate is catalyzed by hydroxycinnamoylglucose:malate hydroxycinnamoyltransferase (HMT; Lehfeldt et al., 2000) and the light requiring conversion of coumaroyl malate to phaselic acid is catalyzed by PPO. It is recognized that due to the complexity of interconversion of secondary metabolites other enzymes may also be involved *in vivo*.

These chloroplastic coumaroyl conjugates can act as substrates for red clover PPO with monophenolase activity. While coumaroyl hexoside was barely detectable in total leaf extracts (data not shown); it was clearly detectable in many chloroplastic extracts, suggesting a localized accumulation perhaps under specific environmental conditions (Figure 6C).

Here, the red clover plants were not visibly stressed, yet removal of leaves after the first of the two phase cross over design could affect plant metabolism during the second phase (via a systemic damage response). It may also remove shading from some of the leaf population. Red clover PPO activity has been shown to increase as young leaves expand and therefore become exposed to higher light intensities (Webb et al., 2013), which might further implicate a role for PPO in protecting the developing photosynthetic apparatus.

The findings reported here are consistent with hydroxylation of the monophenolic coumaroyl conjugate (malate) to a caffeoyl conjugate (malate) in stressed plants in the light. Thus, there may have been conversion of coumaroyl malate to caffeic malate (phaselic acid) by PPO monophenolase in these stressed, illuminated chloroplasts of wild-type plants. Coumaroyl hexoside may be an intermediate metabolite.

In support of this hypothesis is a recently published report on the hypothetical role for walnut (*Juglans regia*) PPO in secondary metabolism (Araji et al., 2014). However, they observed an altered leaf metabolite profile in PPO-silenced walnut compared to the wild-type (Araji et al., 2014), by contrast, no differences between the wild-type and the low leaf PPO mutant population of red clover in terms of phenolic profile were observed in this study (Figure 2).

In conclusion, metabolic profiling has enabled the identification of compounds with the potential to be chloroplast localized PPO substrates. While the hydroxycinnamic acid conjugates require further functional testing, their presence in red clover chloroplasts indicates that in undamaged green tissues, secondary metabolism in chloroplasts could be a neglected component of abiotic stress tolerance. The identification of coumaroyl hexoside and coumaroyl malate in chloroplasts and its apparent conversion to caffeic malate (phaselic acid) in chloroplasts of stressed leaves in the light further highlights the need to continue our efforts in understanding the complexity of secondary metabolism and compartmentation in relation to a role for PPO in undamaged tissues. If PPO activity has a role in protection of the photosynthetic apparatus against damage under abiotic stress conditions, this would make PPO activity a potential breeding target for improved performance of food and bioenergy crops in the future.

## Author contributions

The following authors made substantial contributions to the initial conception and design (AW, KW, AK), to the design, acquisition (TB) and to the analysis and interpretation of the work (TB, AW, KW, AK). The authors have drafted (TB), revised (TB, KW, AW, AK) and given final approval of the work (TB, AW, KW, AK) and all are in agreement to be accountable for all aspects of the work in ensuring that questions related to the accuracy or integrity of any part of the work are appropriately investigated and resolved (TB, AW, KW, AK).

## Funding

This work was supported by IBERS postgraduate studentship (TB), Aberystwyth University (KW), BBSRC (BBS/E/W/10964A01; AK), and BEACON (Welsh European Funding Office; AW).

### Conflict of interest statement

The authors declare that the research was conducted in the absence of any commercial or financial relationships that could be construed as a potential conflict of interest.

## Abbreviations

PPO: polyphenol oxidase
ROS: reactive oxygen species
DOPA: L-3,4-dihydroxyphenylalanine.
